# Parameters of Bone and Cardiovascular Health Related to 25-Hydroxyvitamin D Status in Emirati Nationals attending Primary Care and Diabetes services: a retrospective cohort study

**DOI:** 10.1038/s41598-019-40523-8

**Published:** 2019-03-07

**Authors:** Adam J. Buckley, Maha T. Barakat, Michael F. Holick, Nader Lessan

**Affiliations:** 1 Imperial College London Diabetes Centre, Research Department, Abu Dhabi, United Arab Emirates; 2 Boston University Medical Center, Section of Endocrinology, Diabetes, and Nutrition, Department of Medicine, Boston, MA USA

## Abstract

Vitamin D deficiency is endemic in people living in the Gulf states. We performed a retrospective analysis of data gathered at the first attendance of 82,396 Emirati nationals to outpatient diabetes, endocrinology and general primary care services at two centres in the United Arab Emirates during 2012–2016. Our aim was to explore associations between vitamin D status and markers of cardiovascular and bone health. In the study population, 67.1% of men and 73.5% of women had serum 25(OH)D of less than 50 nmol/L, with the lowest levels being found in young adults. Among Emirati adults with type 2 diabetes, serum 25(OH)D < 50 nmol/L was associated with an increased risk of a coexisting adverse total cholesterol:HDL (TC:HDL) ratio (odds ratio 2.13 (1.60–2.84), p < 0.001). Correcting for age, sex, body mass index, HbA1c and statin therapy, an increase in 25(OH)D of 1 nmol/L was associated with a 0.01 unit reduction in TC:HDL in this population. In a subset of 1064 adult individuals, 25(OH)D < 25 nmol/L was associated with a reduction in DEXA-measured z-score of −0.29 (−0.44 to −0.15, p < 0.001) at the femoral neck and of −0.25(−0.45 to −0.05, p = 0.015) at L1–4, corrected for body mass index, compared with individuals with 25(OH)D ≥ 75 nmol/L. Our findings raise concerns regarding lifetime burden of cardiovascular disease and bone health for young Emiratis with vitamin D deficiency.

## Introduction

Incident UVB-spectrum sunlight available in the United Arab Emirates (UAE) is sufficient to permit adequate previtamin D3 synthesis throughout the year^1^. Despite this, deficiency of vitamin D is highly prevalent in the Arab Emirati population^2^. Although Emirati national dress covers the arms and legs, the face and hands are usually uncovered, and even brief daily exposure of 5% of body surface area to the sun should be sufficient to maintain vitamin D stores^3^. A culture of sun avoidance has been identified as an important factor predisposing to vitamin D deficiency in the UAE, particularly in young adult Emiratis who express the belief that exposure to sunlight is harmful to health^4,5^. The high prevalence of vitamin D deficiency reported in young adult Emiratis occurs despite a good understanding among local health professionals of its importance in maintaining bone health in children. Children born in the UAE are routinely prescribed supplementation with 400 international units of vitamin D3 per day until six months of age, but subsequent vitamin D supplementation is voluntary or through fortification of foods. Dietary vitamin D intake alone is, however, known to frequently be inadequate to meet requirements^6,7^.

Vitamin D deficiency is conventionally defined as a serum 25(OH)D concentration of <50 nmol/l (20 ng/ml), based on the levels at which bone-related symptoms become apparent, and insufficiency as a serum 25(OH)D of 50–75 nmol/l (20–30 ng/ml), determined by the level of vitamin D repletion sufficient for optimum bone health^8,9^. While severe vitamin D deficiency, defined as serum 25(OH)D < 25 nmol/L (10 ng/mL), is unequivocally linked with childhood rickets and adult osteomalacia^10^, the clinical impact of milder forms of deficiency and insufficiency, and the prevalence of true deficiency, are the subject of controversy^11^. Serum 25(OH)D level reflects vitamin D intake and total body vitamin D stores, and is therefore considered to be suitable and sufficient for clinical assessment of vitamin D status^8,12^.

A wide range of tissues express the vitamin D receptor and vitamin D-responsive elements are present in the promoter regions of over 2000 genes^13,14^. This has led to speculation that vitamin D status might affect other aspects of homeostasis and metabolism including neoplasia^15^, hypertension^16^, β-cell function^17^, insulin resistance^18^, and lipid profile^19^. Both Vitamin D deficiency^20–22^ and atherosclerotic heart disease^23^ have been reported to be of high prevalence in the Middle East. We hypothesised that vitamin D status and cardiovascular health are positively associated. We also speculated that vitamin D deficiency would be associated with poorer bone health in our population. We have therefore investigated the demographic associations of 25(OH)D sufficiency in the large population of Emirati individuals attending the ICLDC, with particular emphasis on the potential for interaction with other biochemical and metabolic parameters, bone health, and cardiovascular risk.

## Methods

### Study design and setting

The study was based at the two branches of Imperial College London Diabetes Centre (ICLDC), located in the cities of Abu Dhabi and Al Ain in the United Arab Emirates. ICLDC operates Diabetes, General Endocrinology and Primary Care clinics, with additional specialist clinics in Cardiology and Renal Medicine. Patients attend ICLDC from throughout the Emirates and are predominantly of Arab Emirati origin. Anonymized data from electronic patient records were analysed retrospectively.

### Data acquisition and consent

Data were collected from electronic records of patients enrolled at Imperial College London Diabetes Centre (ICLDC). The study followed the ethical guidelines for retrospective studies, approved by the ICLDC Research Ethics Committee. Informed consent for use of clinical data in anonymised form was obtained from the patients at the time of enrolment. Investigations, including vitamin D status and lipid profile are requested as per clinical need and at discretion of the clinician taking care of individual patient. Every measurement of serum 25(OH)D determined in a patient attending ICLDC for the first time between 01/01/2010 and 30/12/2016 (inclusive) was included in the data set. Participants were assigned to subgroups by vitamin D sufficiency status based on serum 25(OH)D level. These groups were defined according to cut-offs commonly used in clinical practise, with severe deficiency of vitamin D defined as serum 25(OH)D < 25 nmol/l, deficiency as 25–49 nmol/L, insufficiency as 50–74 nmol/l and sufficiency as ≥75 nmol/l. Contemporaneous data regarding body mass index (BMI), date of birth, HbA1c, lipid profile, and the use of vitamin D supplementation or active forms of vitamin D were collected for each individual from their electronic record. Laboratory tests were performed using Elecsys Vitamin D total II kit for Cobas platform (Roche Diagnostics, Indianapolis, Indiana). Inter- and intra- assay variability were 10.7% and 4.6%, respectively.

Additionally, we identified electronic records for 5386 DEXA bone densitometry investigations performed using the Lunar Prodigy Advance system (GE Healthcare, Chicago, IL), between 2014-01-05 and 2016-12-29. Mean Z-scores for L1–L4 and femoral neck were acquired by hand for all individuals with recorded serum 25(OH)D < 25 nmol/L (n = 282). The remaining participants were stratified by 10-year age groups and a further 782 records were selected at random from within each group with the aim of obtaining records from equal numbers of individuals in each age range, where possible, giving a sample size of n = 1064.

### Inclusion and exclusion criteria

All individuals of Emirati origin irrespective of the glucose tolerance status, who attended ICLDC for the first time between 2010-11-11 and 2016-12-31, and in whom serum 25(OH)D status was assessed, were included. Individuals diagnosed with secondary diabetes or monogenic diabetes were excluded from analysis because of the relatively tiny size of these groups. No limitation was placed on age or body habitus.

### Statistical analysis

Data were processed using Microsoft Excel version 15.31 (Microsoft, Redmond) and graphing and statistical analysis were performed using the R Language for Statistical Computing version 3.4 (R Core Team, Vienna, Austria)^24^. Where parameters were skewed, they were log-transformed for inclusion in regression analyses. Correcting for multiple statistical tests and assuming a total of fifty tests would be performed, the threshold for significance was set at p = 0.001.

## Results

### 25(OH)D status in the study population

**Figure 1:**
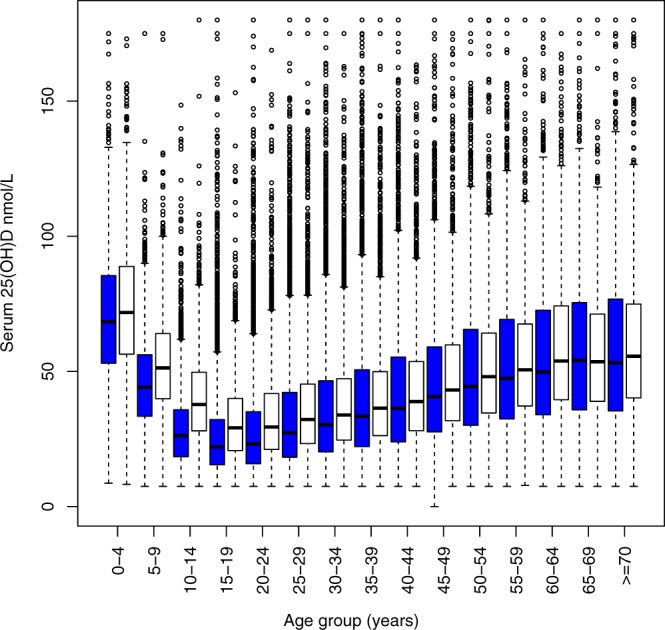
Variation in serum 25(OH)D in all included individuals stratified by age group and sex; white bars represent males, blue bars represent females. Tukey plots represent median, interquartile range (IQR), 25th percentile −1.5 × IQR and 75th percentile +1.5 * IQR.

The baseline characteristics of the 81598 participants overall and by categories of 25(OH)D are summarised in Table 1. The overall prevalence of serum 25(OH)D < 25 nmol/L was 28.8% (Males: 19.9%, Females: 34.2%). A further 42.2% (Males: 47.2%, Females: 39.2%) presented with serum 25(OH)D between 25 and 50 nmol/L. In total, 67.1% of Emirati men and 73.5% of Emirati women presented with serum 25(OH)D of <50 nmol/L. Vitamin D supplements were frequently prescribed, with 69.9% of patients already taking cholecalciferol or ergocalciferol equivalent to at least 400 international units per day at the first presentation. The relationships between serum 25(OH)D, age, and sex are illustrated in Fig. 1. Adjusting for the month in which sampling occurred, serum 25(OH)D decreased by 3.1 (3.1 to 3.2) nmol/L per year between infancy and age 18, while male sex was associated with a 6.4 (5.9 to 7.0) nmol/L higher serum 25(OH)D (all p < 0.001, adjusted R^2^ 0.42; see Supplementary Table 1).

**Table 1:** Summary of participant characteristics for 81598 individuals included in the study as a whole and grouped by vitamin D status.

25(OH)D	<25 nmol/L	25–49 nmol/L	50–74 nmol/L	≥75 nmol/L	All
Sex (M/F)	6124/17397	14528/19928	6826/8514	3318/4963	30796/50802
Age (Years)	29.0 ± 12.3	32.4 ± 16.3	34.2 ± 20.5	37.2 ± 22.4	32.3 ± 17.1
NGT	15164 (31.4%)	20097 (41.6%)	8621 (17.8%)	4426 (9.2%)	48308
Prediabetes	5118 (29.9%)	7590 (44.4%)	2985 (17.5%)	1403 (8.2%)	17096
Type 1	278 (23.7%)	489 (41.7%)	279 (23.8%)	128 (10.9%)	1174
Type 2	2961 (19.7%)	6280 (41.8%)	3455 (23%)	2324 (15.5%)	15020
Ca^2+^	2.23 ± 0.08	2.25 ± 0.08	2.26 ± 0.09	2.28 ± 0.1	2.25 ± 0.09
PTH	5.85 (4.53–7.63)	5.18 (4.02–6.63)	4.76 (3.74–6.09)	4.39 (3.40–5.65)	5.21 (4.03–6.78)
ALP	84.0 (66.2–116.35)	80.0 (63.1–117.0)	73.6 (59–97.8)	68.8 (56–85.27)	77.8 (61.8–107.0)
HDL	1.31 ± 0.34	1.32 ± 0.35	1.34 ± 0.36	1.37 ± 0.37	1.32 ± 0.35
TC	4.47 ± 0.95	4.64 ± 0.99	4.63 ± 0.99	4.58 ± 0.99	4.58 ± 0.98
TC:HDL	3.38 (2.75–4.23)	3.5 (2.83–4.42)	3.43 (2.81–4.31)	3.34 (2.74–4.15)	3.44 (2.79–4.32)
TG	0.91 (0.65–1.35)	1.01 (0.71–1.49)	1.05 (0.74–1.51)	1.07 (0.77–1.50)	0.99 (0.70–1.45)

25(OH)D = serum 25(OH)D, NGT = normal glucose tolerance, “Prediabetes” refers to impaired fasting glucose, impaired glucose tolerance or gestational diabetes, Ca^2+^  = corrected calcium, PTH = parathyroid hormone, ALP = alkaline phosphatase, HDL = high-density lipoprotein, TC = total cholesterol, TG = triglycerides.

Adjusting for the effects of body mass index, sex, glycaemic status and month of sampling on serum 25(OH)D in Emirati adults aged ≥18 year, each one year of age was associated with an increase in serum 25(OH)D of 0.68 (0.67 to 0.7) nmol/L, while each 1 kg/m^2^ increase in body mass index was associated with a reduction in serum 25(OH)D of 0.35 (0.38 to 0.32) nmol/L. Male sex was associated with a 2.34 (1.94 to 2.73) nmol/L increase in serum 25(OH)D. The presence of a prediabetic state or type 2 diabetes were associated with reductions in 25(OH)D of 1.98 (1.5 to 1.98) nmol/L, and 1.85 (1.27 to 2.43) nmol/L, respectively, when compared with normal glucose tolerance (p < 0.001). Type 1 diabetes was not associated with a significant change in serum 25(OH)D. The overall model fit was poor, with the covariates together accounting for 13.2% of the variation seen in serum 25(OH)D (Supplementary Table 2).

### 25(OH)D status, PTH and bone profile in the study population

Serum parathyroid hormone (PTH), corrected calcium (Ca2+), phosphate (PO4) and alkaline phosphatase (ALP) were measured in a subset of the population aged >20 years; younger individuals were excluded due to the effects of growth on ALP and PO4. Median values for each parameter are presented in Table 2 along with the number of participants with available information. Kruskal-Wallis tests indicated significant differences in each parameter according to vitamin D status (p < 0.001 for each). Pairwise Mann-Whitney U tests with Bonferroni correction for multiple comparisons were performed to determine the significance of differences between groups. PTH was significantly lower in the individuals with serum 25(OH)D of ≥75 nmol/L than in each other group (all p < 0.001), while PTH was also significantly higher in each sufficiency group than the next higher group (25–49 nmol/L cf 50–74 nmol/L p < 0.001, <25 nmol/L cf 25–49 nmol/L p < 0.001).

**Table 2:** Biochemical parameters by 25(OH)D sufficiency status in Emirati individuals aged > 20 years. P-values refer to Kruskal-Wallis test for difference between groups.

25(OH)D	<25 nmol/L	25–49 nmol/L	50–74 nmol/L	≥75 nmol/L	N	Sig.
Ca^2+^	2.22 (2.17–2.27)	2.23 (2.18–2.28)	2.24 (2.19–2.29)	2.26 (2.2–2.32)	52618	<0.001***
PTH	6.01 (4.62–7.83)	5.23 (4.07–6.69)	4.8 (3.77–6.12)	4.42 (3.41–5.68)	40438	<0.001***
ALP	74.4 (61.3–91.1)	71.7 (59.0–87.2)	69.0 (56.6–83.0)	66.6 (55.0–80.7)	24802	<0.001***
PO_4_	1.05 (0.94–1.16)	1.06 (0.95–1.16)	1.06 (0.95–1.17)	1.07 (0.96–1.19)	52619	<0.001***

25(OH)D = serum 25(OH)D, Ca^2+^  = corrected serum calcium (mmol/L), PTH = parathyroid hormone (pmol/L), ALP = alkaline phosphatase (IU/L), PO_4_ = serum phosphate (mmol/L).

Serum Ca^2+^ was significantly higher in Emirati adults with serum 25(OH)D ≥ 75 nmol/L (all p < 0.001, n = 52618,), while serum ALP was significantly lower (all p < 0.001, n = 24802,) than in each of the other vitamin D status groups. Serum phosphate was significantly higher in individuals with serum 25(OH)D ≥ 75 nmol/L than in individuals with serum 25(OH)D ≤ 49 nmol/L (p < 0.001, n = 52619).

DEXA Bone densitometry reports were examined in 1064 individuals, selected as described in the Methods section, and Z-scores based on Western values for age-matched bone mineral density were recorded for mean lumbar spine (L1–L4) and mean femoral neck, along with serum 25(OH)D at first presentation. In male participants (n = 268), mean Z-score for L1–4 was −0.33 ± 1.40 and mean Z-score for femoral neck was −0.24 ± 1.06, while in female participants (n = 796), mean Z-score for L1–4 was −0.51 ± 1.25 and mean Z-score for femoral neck was −0.19 ± 0.88. Z-score is a measure of the number of standard deviations from the age-adjusted mean and therefore, in a healthy population and with suitable reference values, it would be expected that mean Z-score would be 0.0 with standard deviation of 1.0. Correcting for the effect of body mass index using linear regression, serum 25(OH)D was significantly and positively associated with bone mineral density at the femoral neck, albeit with a small effect size. A 10 nmol/L increase in 25(OH)D was associated with an increase in Z score at the femoral neck of 0.01 (p < 0.001; Supplementary Table 3). This result should be interpreted with considerable caution since the models explained only 3% of the variability of z-score. Inclusion of log-transformed PTH in normocalcaemic individuals greatly improved model fit such that 8% of the variability of the Z-score at the femur was explained (Supplementary Table 4), with the significant limitations that, due to missing data, only 508 individuals were included, that 25(OH)D was not a significant predictor in this model, and that PTH reflects both vitamin D status and dietary calcium intake in normocalcaemic individuals.

### 25(OH)D status and cardiovascular risk profile in Emirati adults with type 2 diabetes

**Figure 2:**
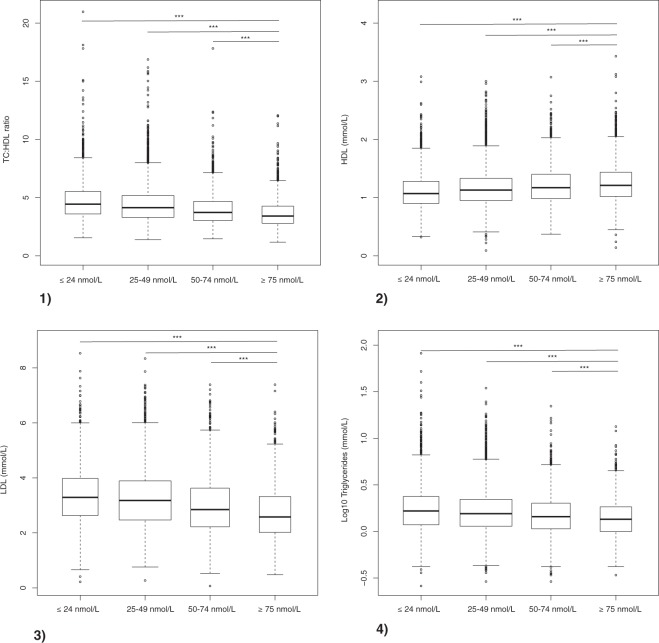
Lipid status by 25(OH)D status in 14559 Emirati individuals with a diagnosis of type 2 diabetes. (1) Total cholesterol: high density lipoprotein (TC:HDL) ratio, four outlying values excluded, (2) Serum HDL (mmol/L), three outlying values excluded, (3) Serum LDL (mmol/L), (4) Log-transformed serum triglycerides (mmol/L). Tukey plots represent median, interquartile range, 25th percentile −1.5 * IQR and 75th percentile +1.5 * IQR. All differences between individual groups were highly significant, (p < 0.0001, multiple Mann-Whitney U tests with correction for multiple comparisons). Only pairwise comparisons between the ≥75 nmol/L group and each other group are included for clarity.

We examined the relationship between vitamin D status and lipid profile in 14559 participants with a diagnosis of type 2 diabetes. Serum 25(OH)D of <50 nmol/L is commonly considered to indicate vitamin D deficiency in routine clinical practice. The ratio of total cholesterol to HDL (TC:HDL) is considered a marker of metabolic health, with higher values denoting increased risk of cardiovascular disease. The median TC:HDL was 3.59 (2.93–4.51) in individuals with serum 25(OH)D < 50 nmol/L and 4.24 (3.39–5.3) in individuals with serum 25(OH)D > 50 nmol/L (p < 0.001). Median TC:HDL ratios, grouped by serum 25(OH)D level, are presented in Fig. 2.

Per data from the Framingham Study^25^, TC:HDL < 3.4 in women or <3.5 in men is associated with a 50% reduction in cardiovascular risk, while TC:HDL ≥ 7.0 in women or ≥9.6 in men is associated with a two-fold increase in cardiovascular risk. The proportions of participants who had favourable or unfavourable lipid profiles are presented in Table 3, grouped by serum 25(OH)D level. Among participants with 25(OH)D < 50 nmol/L, 2448 (26.5%) had an favourable TC:HDL ratio, compared with 2574 (44.6%) in those with 25(OH)D ≥ 50 nmol/L. This difference in proportions was significant (p < 0.001, 2.23 (2.08–2.39), chi-square test). Conversely, individuals with 25(OH)D < 50 nmol/L were significantly more likely to have an unfavourable TC:HDL ratio than those serum 25(OH)D > 50 nmol/L (p < 0.001, OR 2.13 (1.60–2.84), chi-square test).

**Table 3:** Ratio of total cholesterol (TC) to HDL by 25(OH)D status in 14559 Emirati adults with type 2 diabetes mellitus. According to the Framingham study, in women a ratio of ≤3.4 is associated with an approximately 50% reduction in cardiovascular risk while a ratio of ≥7.0 is associated with a two-fold increase in cardiovascular risk; in men the cutoffs are ≤3.5 and ≥9.6.

	TC:HDL ≤3.4 (women) or ≤3.5 (men)	TC:HDL 3.5–6.9 (women) or 3.6–9.5 (men)	TC:HDL ≥7.0 (women) or ≥9.6 (men)
<25 nmol/L	589 (20.5%)	2198 (76.6%)	84 (2.9%)
25–49 nmol/L	1778 (29.1%)	4215 (69.0%)	113 (1.8%)
50–74 nmol/L	1357 (40.6%)	1947 (58.3%)	36 (1.1%)
≥75 nmol/L	1132 (50.5%)	1091 (48.7%)	19 (0.9%)

Correcting for the potential effects of body habitus, glycaemic control, sex, statin treatment, and age on the relationship between TC:HDL ratio and serum 25(OH)D in 14579 individuals with type 2 diabetes in linear regression, a serum 25(OH)D of less than 50 nmol/L was independently associated with an increase in TC:HDL ratio of 0.43 (95% CI 0.38–0.48) (p < 0.001; see Supplementary Table 5**)**. Serum 25(OH)D was negatively associated with TC:HDL (10 nmol/L increase in 25(OH)D associated with a 0.1 unit reduction in TC:HDL, p < 0.001; see Table 4).

**Table 4:** Linear regression of serum 25(OH)D, age, sex, body mass index, HbA1c and use of a statin on TC:HDL ratio in 14559 Emirati adults with a diagnosis of type 2 diabetes.

	β	B	SE	t	p
(Intercept)		4.50	0.11	42.3	<0.001***
25(OH)D	−0.144	−0.01	0.00	−17.70	<0.001***
Age (Years)	−0.187	−0.02	0.00	−21.50	<0.001***
Male sex	0.393	0.62	0.03	24.60	<0.001***
BMI (kg/m^2^)	−0.014	−0.00	0.00	−1.70	0.081
HbA1c (%)	0.158	0.11	0.01	20.20	<0.001***
Statin	0.155	0.24	0.03	9.30	<0.001***

Adjusted R^2^ = 0.157. β = standardized coefficient (change in SD of dependent variable per SD change of independent variable), B = non-standardised coefficient (unit change in dependent variable per unit change in independent variable).

## Discussion

The prevalence and patterns of vitamin D deficiency and insufficiency we found in Emirati nationals attending outpatient services were comparable with reports from the Middle East and North Africa (MENA) area. Between 30.7 and 61.8% of residents in the Lebanon, and 50–63% of residents of Jordan are reported to be deficient or insufficient in vitamin D^20^. 72.1% of a population of female students attending a high school in Iran were reported to have vitamin D deficiency^21^, while 81% of female students aged 12–15 attending a school in the Kingdom of Saudi Arabia were reported to have serum 25(OH)D of less than 25 nmol/L^22^. Only a limited number of studies have specifically investigated the prevalence of, and local factors associated with, vitamin D deficiency in the United Arab Emirates. One study conducted in Al Ain demonstrated hypovitaminosis D in 65.1% of a population of 143 adolescents, with vitamin D deficiency ≤ 50 nmol/l in 10% of boys and 28% of girls^26^. Vitamin D status was found to be paradoxically lower in summer than in winter in a population of 138 female university students in Abu Dhabi, attributed to high levels of sun avoidance and high outside temperatures; sun avoidance was independently negatively correlated with serum 25(OH)D levels^4^. 31.2% of children aged 8–14 attending an ambulatory clinic at Sheikh Khalifa Medical City in Abu Dhabi had serum 25(OH)D measured at <25 nmol/l^27^. In 141 indoor workers resident in the UAE, median 25(OH)D was reported as 22.4 nmol/l, with 63.2% being severely deficient and 29.1% deficient in vitamin D, and serum 25(OH)D was correlated with a 5-point scoring system rating sun-avoidant behaviours^28^. In the Emirati population we studied, 71.05% presented with serum 25(OH)D < 50nmol/l.

Our findings support the conclusion that, although the clinical impact of vitamin D insufficiency and moderate deficiency remains the source of some debate, serum 25(OH)D below 75 nmol/L is associated with small but statistically significant differences in serum parathyroid hormone, corrected calcium and alkaline phosphatase. In keeping with this finding, lower serum vitamin D was associated with a reduction in age-adjusted bone mineral density at femoral neck when corrected for body mass index. It remains possible that the coexisting clinical indications warranting investigation in the subset of individuals who underwent bone densitometry may have masked the impact of vitamin D status. Previous studies reports a lack of concordance between densitometry and vitamin D status in Hungarian men^29^ and American men of black or Hispanic origin^30^, although an association between bone mineral density at the hip and vitamin D status was found in a multinational cohort of postmenopausal women^31^. Comprehensive reference ranges for bone mineral density are not available for the Emirati population, and a PubMed search did not identify any studies specifically exploring the association between vitamin D status and bone mineral density in Arabic populations.

Our finding that low serum 25(OH)D is associated with a less favourable lipid profile is consistent with the previously published results of observational studies. In 107,811 American patients, 25(OH)D < 50 nmol/L was associated with lower HDL, higher LDL, higher total cholesterol and higher triglycerides^32^. Plasma 25(OH)D was positively associated with HDL in 237 children and adolescents living in the United States^33^. Low circulating 25(OH)D has been associated with an increased risk of myocardial infarction in men and with increased prevalence of cardiovascular disease in adult men and women^34,35^. Vitamin D supplementation has not, however, yet been demonstrated to provide beneficial effects on lipid profile and cardiovascular outcomes, although the randomized controlled trials performed to date have been relatively small and heterogeneous in their interventions and study populations^36^.

The relationship of 25(OH)D to age in our study population suggests that, although vitamin D supplementation for infants is effective, vitamin D status declines rapidly in childhood and young adulthood, especially young women. The most plausible explanation for this would appear to be sun avoidance in this population, while the increase in median 25(OH)D with age in older adults may be explained by differing attitudes to sun exposure and to incidental identification of vitamin D deficiency during treatment for other conditions. This raises two concerns for population health, which require further study. Firstly, adequate vitamin D status in pregnant women is important because of the associated risk of neonatal hypocalcaemia and tetany^37^, which is particularly prevalent in women with diabetes mellitus due to associated prematurity, hypomagnesemia and hyperparathyroidism^38^. Secondly, the association between vitamin D deficiency and an unfavourable lipid profile is cause for concern because of the possibility that young people who are vitamin D deficient could suffer from an increased risk of cardiovascular disease later in life. Diseases of the circulatory system are already the most common cause of death in Emirati nationals, accounting for 35% of deaths in 2015^2^.

Owing to the retrospective nature of our study, there are certain limitations and potential sources of bias. Due to the extremely large size of the data set, it was necessary to choose a single attendance from several within the time period, and data regarding each individual’s diagnosis and treatment prior to attendance at ICLDC were not accessible electronically. The median age of participants was 43.7 years, which is older than the estimated overall median age of 30 years of the Emirati population. The included participants were patients attending a clinic rather than being randomly selected, which would increase the prevalence of coexisting illnesses, treatments, and other confounders. Due to the specialization of ICLDC, a large proportion of the sample consisted of people with type 2 diabetes; the International Diabetes Federation estimates the prevalence of diabetes mellitus in adults resident in the UAE at 14.6%, whereas 18.4% of the study population were diagnosed with type 2 diabetes, 1.4% with type 1 diabetes, 21.0% with impaired fasting glucose or impaired glucose tolerance and 59.2% with normal glucose tolerance. However, one strength of the study design is the large proportion of all Emirati individuals included; Health Authority Abu Dhabi (HAAD) estimated the total number of Emirati Nationals living in the UAE in 2014 at 524,023^2^, and consequently the data set presented here represents up to 15.6% of the entire population.

To conclude, Vitamin D deficiency is highly prevalent in Emirati nationals attending primary care services. The effects on biochemical parameters including lipid profile raise concerns regarding a possible effect on the future cardiovascular health of the United Arab Emirates. Longitudinal research regarding the effectiveness of vitamin D supplementation on lipid profile and cardiovascular risk in the Emirati population is needed.

## Supplementary information

Supplementary files
