# Supplementary material for: Parameters of Bone and Cardiovascular Health Related to 25-Hydroxyvitamin D Status in Emirati Nationals attending Primary Care and Diabetes services: a retrospective cohort study
Source: Sci Rep. 2019 Mar 7;9:3835. doi: 10.1038/s41598-019-40523-8 (PMC6405844; doi:10.1038/s41598-019-40523-8)
Supplement: Supplementary file 1 — Supplementary files [file 41598_2019_40523_MOESM1_ESM.docx]

**Parameters of Bone and Cardiovascular Health Related to 25-Hydroxyvitamin D Status in Emirati Nationals attending Primary Care and Diabetes services: a retrospective cohort study**

Adam J Buckley1, Maha T Barakat1, Michael F Holick2 and Nader Lessan1*

**1** Imperial College London Diabetes Centre, Research Department (Abu Dhabi, United Arab Emirates)

**2** Boston University Medical Center, Section of Endocrinology, Diabetes, and Nutrition, Department of Medicine, Boston, MA, USA

Correspondence to:

Nader Lessan MD, FRCP

Consultant Endocrinologist

Imperial College London Diabetes Centre

PO Box 48338

Abu Dhabi, United Arab Emirates

Telephone: 009712 4040800 Ext 928

Fax: 009712 4040900

Email: [nlessan@icldc.ae](mailto:nlessan@icldc.ae)

**Supplementary Tables**

**Supplementary Table 1:** Linear regression of age, sex and month of sampling on serum vitamin D in 17707 Emirati individuals aged < 18 years. Adjusted R^2^ = 0.42. β = standardized coefficient (change in sd of dependent variable per sd change of independent variable), B = non-standardised coefficient (unit change in dependent variable per unit change in independent variable)

|  | β | B | SE | t | p |
| --- | --- | --- | --- | --- | --- |
| (Intercept) |  | 70.87 | 0.62 | 114.01 | <0.001 *** |
| Age (years) | -0.61 | -3.15 | 0.03 | -106.01 | <0.001 *** |
| Male Sex | 0.27 | 6.41 | 0.28 | 22.83 | <0.001 *** |
| February | -0.02 | -0.56 | 0.76 | -0.73 | 0.464 |
| March | 0.09 | 2.12 | 0.70 | 3.02 | 0.003 ** |
| April | 0.08 | 1.85 | 0.65 | 2.84 | 0.005 ** |
| May | 0.12 | 2.98 | 0.73 | 4.09 | <0.001 *** |
| June | 0.08 | 1.88 | 0.74 | 2.54 | 0.011 * |
| July | 0.16 | 3.97 | 0.73 | 5.41 | <0.001 *** |
| August | 0.20 | 4.87 | 0.66 | 7.43 | <0.001 *** |
| September | 0.11 | 2.73 | 0.75 | 3.66 | <0.001 *** |
| October | 0.13 | 3.24 | 0.72 | 4.54 | <0.001 *** |
| November | 0.18 | 4.34 | 0.73 | 5.98 | <0.001 *** |
| December | 0.15 | 3.70 | 0.68 | 5.46 | <0.001 *** |

**Supplementary Table 2:** Linear regression of age, body mass index, glycaemic status, sex and month of sampling on serum vitamin D in 63891 Emirati individuals aged ≥ 18 years. Adjusted R^2^ = 0.13. β = standardized coefficient (change in sd of dependent variable per sd change of independent variable), B = non-standardised coefficient (unit change in dependent variable per unit change in independent variable)

|  | β | B | SE | t | p |  |
| --- | --- | --- | --- | --- | --- | --- |
| (Intercept) |  | 22.20 | 0.61 | 36.39 | < 0.001 | *** |
| Age (years) | 0.38 | 0.68 | 0.01 | 82.45 | < 0.001 | *** |
| BMI (kg/m2) | -0.09 | -0.35 | 0.02 | -23.39 | < 0.001 | *** |
| Prediabetes | -0.08 | -1.98 | 0.25 | -8.02 | < 0.001 | *** |
| Type 1 diabetes | 0.07 | 1.68 | 1.02 | 1.65 | 0.100 | . |
| Type 2 diabetes | -0.07 | -1.85 | 0.30 | -6.22 | < 0.001 | *** |
| Male sex | 0.09 | 2.34 | 0.20 | 11.67 | < 0.001 | *** |
| February | -0.10 | -0.24 | 0.49 | -0.50 | 0.618 |  |
| March | 0.08 | 1.97 | 0.47 | 4.17 | < 0.001 | *** |
| April | 0.06 | 1.65 | 0.46 | 3.56 | < 0.001 | *** |
| May | 0.15 | 3.89 | 0.47 | 8.30 | < 0.001 | *** |
| June | 0.16 | 4.04 | 0.49 | 8.20 | < 0.001 | *** |
| July | 0.21 | 5.38 | 0.54 | 9.92 | < 0.001 | *** |
| August | 0.21 | 5.40 | 0.48 | 11.28 | < 0.001 | *** |
| September | 0.15 | 3.84 | 0.47 | 8.13 | < 0.001 | *** |
| October | 0.15 | 3.83 | 0.47 | 8.28 | < 0.001 | *** |
| November | 0.14 | 3.60 | 0.47 | 7.63 | < 0.001 | *** |
| December | 0.10 | 2.47 | 0.48 | 5.19 | < 0.001 | *** |

**Supplementary Table 3:** Linear regression of serum 25(OH)D and BMI on Z-score for femoral neck in 1064 Emirati individuals. The model fit is poor, adjusted R^2^ = 0.03. β = standardized coefficient (change in sd of dependent variable per sd change of independent variable), B = non-standardised coefficient (unit change in dependent variable per unit change in independent variable)

|  | **β** | **B** | **SE** | **t** | **p** |  |
| --- | --- | --- | --- | --- | --- | --- |
| **(Intercept)** |  | -1.110 | 0.163 | -6.85 | < 0.001 | *** |
| **25(OH)D** | 0.127 | 0.001 | 0.001 | 4.09 | < 0.001 | *** |
| **BMI** | 0.155 | 0.020 | 0.010 | 4.99 | < 0.001 | *** |

**Supplementary Table 4:** Linear regression of serum 25(OH)D, BMI and log-transformed PTH on Z-score for femoral neck in 508 Emirati individuals. The model fit is poor, adjusted R2 = 0.08. β = standardized coefficient (change in sd of dependent variable per sd change of independent variable), B = non-standardised coefficient (unit change in dependent variable per unit change in independent variable)

|  | β | B | SE | t | p |  |
| --- | --- | --- | --- | --- | --- | --- |
| (Intercept) |  | -0.407 | 0.252 | -1.613 | 0.107 |  |
| 25(OH)D | 0.058 | 0.002 | 0.001 | 1.322 | 0.187 |  |
| BMI (kg/m2) | 0.151 | 0.024 | 0.006 | 3.667 | < 0.001 | *** |
| log10 PTH (pmol/L) | -0.241 | -0.910 | 0.174 | -5.226 | < 0.001 | *** |

**Supplementary Table 5:** Linear regression of vitamin D status, age, sex, body mass index, HbA1c and use of a statin on TC:HDL ratio in 14559 Emirati adults with a diagnosis of type 2 diabetes. Adjusted R^2^ = 0.152. β = standardized coefficient (change in sd of dependent variable per sd change of independent variable), B = non-standardised coefficient (unit change in dependent variable per unit change in independent variable)

|  | β | B | SE | t | p |
| --- | --- | --- | --- | --- | --- |
| (Intercept) |  | 3.84 | 0.11 | 36.37 | < 0.001 *** |
| 25(OH)D < 50 nmol/L | 0.27 | 0.43 | 0.03 | 16.41 | < 0.001 *** |
| Age (Years) | -0.19 | -0.02 | 0.00 | -22.48 | < 0.001 *** |
| Male Sex | 0.39 | 0.62 | 0.03 | 24.64 | < 0.001 *** |
| BMI (kg/m2) | -0.01 | -0.00 | 0.00 | -1.31 | 0.192 |
| HbA1c (%) | 0.16 | 0.11 | 0.01 | 20.56 | < 0.001 *** |
| Statin | 0.16 | 0.25 | 0.03 | 9.33 | < 0.001 *** |
